# Melatonin: highlighting its use as a potential treatment for SARS-CoV-2 infection

**DOI:** 10.1007/s00018-021-04102-3

**Published:** 2022-02-20

**Authors:** Russel J. Reiter, Ramaswamy Sharma, Fedor Simko, Alberto Dominguez-Rodriguez, Jan Tesarik, Richard L. Neel, Andrzej T. Slominski, Konrad Kleszczynski, Verna M. Martin-Gimenez, Walter Manucha, Daniel P. Cardinali

**Affiliations:** 1 Department of Cell Systems and Anatomy, UT Health San Antonio, Long School of Medicine, San Antonio, TX USA; 2 Institute of Pathophysiology, Faculty of Medicine, Comenius University, Bratislava, Slovak Republic; 3 Department of Cardiology, Hospital Universitario de Canarias, Santa Cruz de Tenerife, Spain; 4 MARGen Clinic, 18006 Granada, Spain; 5 CEO, Alcasian Care Enterprises, Castroville, TX USA; 6 Department of Dermatology, University of Alabama at Birmingham, Birmingham, AL USA; 7 Pathology and Laboratory Medicine Service, VA Medical Center, Birmingham, AL USA; 8 Department of Dermatology, University of Münster, Münster, Germany; 9 Instituto de Investigaciones en Ciencias Químicas, Facultad de Cs. Químicas Y Tecnológicas, Universidad Católica de Cuyo, San Juan, Argentina; 10 Área de Farmacología, Departamento de Patología, Facultad de Ciencias Médicas, Universidad Nacional de Cuyo, Mendoza, Argentina; 11 Faculty of Medical Sciences, Pontificia Universidad Católica Argentina, Buenos Aires, Argentina

**Keywords:** Cytokine storm, COVID-19, Viral infection, Coronavirus, Sepsis, Hypoxia-inducible factor 1-α, Phospholipase A2

## Abstract

Numerous pharmaceutical drugs have been repurposed for use as treatments for COVID-19 disease. These drugs have not consistently demonstrated high efficacy in preventing or treating this serious condition and all have side effects to differing degrees. We encourage the continued consideration of the use of the antioxidant and anti-inflammatory agent, melatonin, as a countermeasure to a SARS-CoV-2 infection. More than 140 scientific publications have identified melatonin as a likely useful agent to treat this disease. Moreover, the publications cited provide the rationale for the use of melatonin as a prophylactic agent against this condition. Melatonin has pan-antiviral effects and it diminishes the severity of viral infections and reduces the death of animals infected with numerous different viruses, including three different coronaviruses. Network analyses, which compared drugs used to treat SARS-CoV-2 in humans, also predicted that melatonin would be the most effective agent for preventing/treating COVID-19. Finally, when seriously infected COVID-19 patients were treated with melatonin, either alone or in combination with other medications, these treatments reduced the severity of infection, lowered the death rate, and shortened the duration of hospitalization. Melatonin’s ability to arrest SARS-CoV-2 infections may reduce health care exhaustion by limiting the need for hospitalization. Importantly, melatonin has a high safety profile over a wide range of doses and lacks significant toxicity. Some molecular processes by which melatonin resists a SARS-CoV-2 infection are summarized. The authors believe that all available, potentially beneficial drugs, including melatonin, that lack toxicity should be used in pandemics such as that caused by SARS-CoV-2.

## Introduction

Since the classification of SARS-CoV-2 infection as a pandemic, in excess of 140 publications have urged consideration of melatonin as a safe and potentially effective treatment for this worldwide disease [1–13]. The rationale for its use not only stems from its high safety profile [14–16] but also from its multiple beneficial actions in experimental and clinical studies related to the pandemic. This endogenously produced molecule is a broad-spectrum antiviral agent [17, 18] that has shown efficacy in reducing the severity of COVID-19. Yet, the idea of its use has not generated any desirable interest at the governmental or pharmaceutical level. Meanwhile, numerous potentially toxic and expensive repurposed drugs have been espoused or used as clinical treatments, e.g., colchicine [19], glucocorticoids [20], remdesivir [14, 21], and many others [22–25]. Although of significant value, even the currently available vaccines are not without occasional serious side effects [26–28]. Moreover, the efficacy of the vaccines has decreased as the virus has mutated [29, 30]. Already, there are several variants that have been identified and there will likely be others that may further reduce the effectiveness of the vaccines. Summarized in this report are some actions of melatonin that support its use in the prevention and/or treatment of SARS-CoV-2 infections.

The search terms used to identify the published literature related to melatonin and its potential association with COVID-19/SARS-CoV-2 are summarized in Table 1. The searches used PubMed.gov and were performed on November 21, 2021. Among these reports, publications related to the ability of melatonin to suppress inflammation and the cytokine storm associated with COVID-19 disease were most frequent. Melatonin use as a treatment for humans infected with SARS-CoV-2 also was a common consideration. A summary of the outcomes/endpoints of the studies/clinical trials in which melatonin was used as a treatment or in which network analysis suggested the high likelihood of melatonin being an effective treatment for COVID-19 is discussed in the appropriate section below (See Effective Dose, Timing of Administration, Limitations, and Future Studies). The already-published data confirming the efficacy of melatonin in reducing the severity of human COVID-19 infection are summarized in tabulator form later in this report.

**Table 1:** Results (number of related publications) of the search terms using Pubmed.gov which relate to COVID-19 and melatonin

Search terms	Number of publications
COVID-19, melatonin	143
COVID-19, melatonin, inflammation	47
COVID-19, melatonin, cytokine storm	30
COVID-19, melatonin, ARDS*	12
COVID-19, melatonin, sepsis	8
COVID-19, melatonin, anosmia, ageusia	1
COVID-19, melatonin, aging	10
COVID-19, melatonin, human	107
COVID-19, melatonin, treatment	97
COVID-19, melatonin, mechanisms	25

When SARS-CoV-2 was used in lieu of COVID-19, similar results were obtained. Searches were conducted on November 21, 2021

^*^ARDS Acute Respiratory Distress Syndrome

## Melatonin and sepsis

**Fig. 1:**
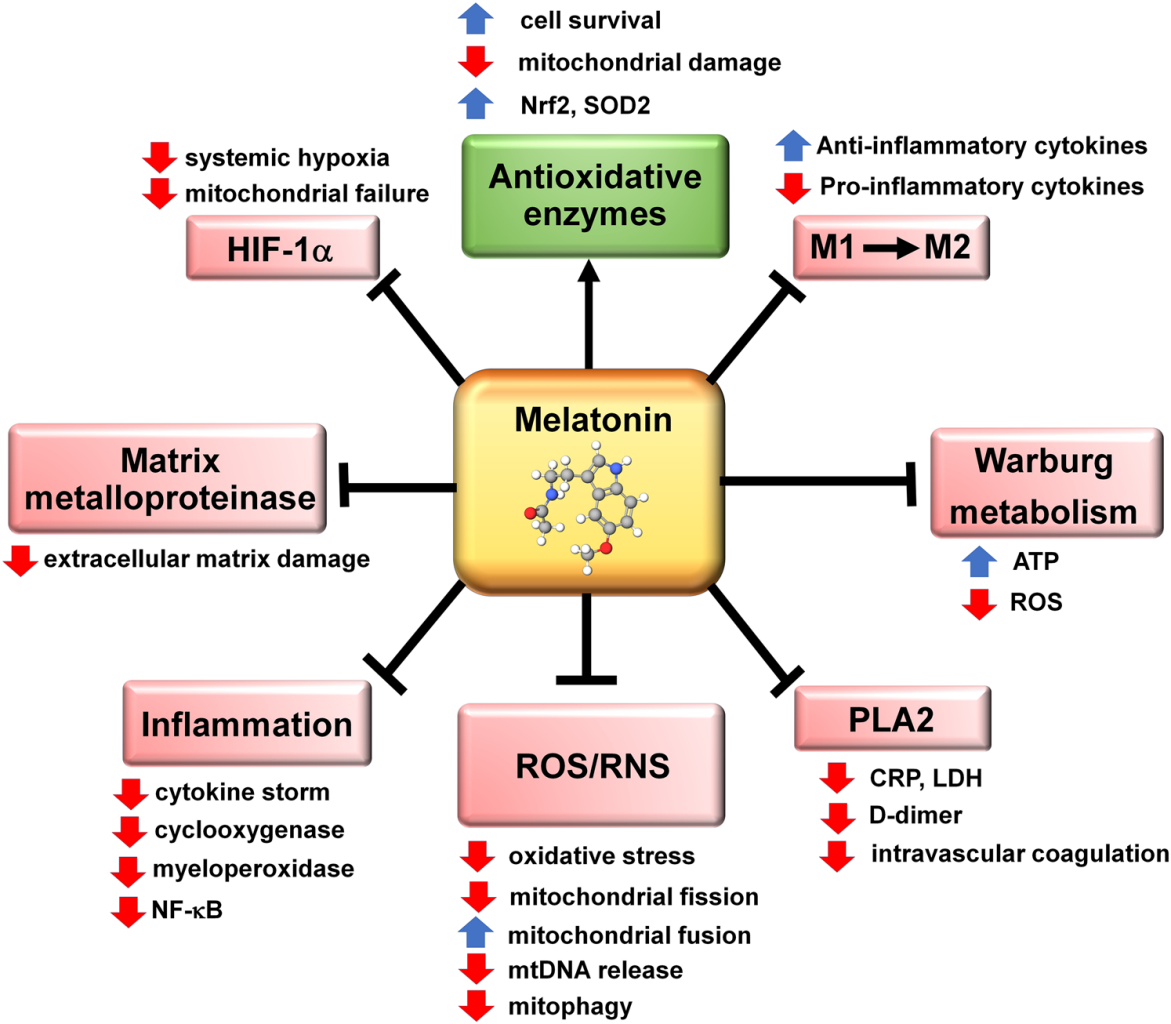
The actions of melatonin that contribute to the ability of this ubiquitously distributed molecule in reducing the severity of a SARS-CoV-2 infection. HIF-1α = hypoxia-inducible factor 1 alpha; M1 = pro-inflammatory macrophage; M2 = anti-inflammatory macrophage; mtDNA = mitochondrial DNA; NF-ҝB = nuclear factor kappa B; Nrf2 = transcription factor NF-E2 p45 transcription factor; PLA2 = phospholipase A2; RNS = reactive nitrogen species; ROS = reactive oxygen species; SOD2 = superoxide dismutase 2. Pointed arrow = stimulation; blunt arrows = inhibition

While SARS-CoV-2 infections are generally thought of as a pulmonary issue, the consequences of this infection transcend the respiratory system. Ultimately, this disease becomes systemic with the development of severe sepsis or septic shock leading to multiple organ failure which is the condition that commonly leads to death of SARS-CoV-2-infected patients. Sepsis can occur as a consequence of a viral, bacterial, or fungal infection and, from a pathophysiological perspective, the damage to multiple organs leading to their failure and death of the patient has the same cause, i.e., the cytokine storm and hyperinflammation with extensive oxidative damage [17, 31–33]. Due to is potent antioxidant and anti-inflammatory effects, melatonin has frequently been proposed for use to overcome the cytokine storm in virus-related infections [34, 35], including that caused by SARS-CoV-2 [36–38]) (Fig. 1).

In bacteria-related sepsis as well, melatonin is an effective prophylactic agent [39, 40]. Importantly, melatonin prevented the death of premature newborn humans suffering from severe bacterial sepsis or septic shock [41] and melatonin has been suggested for use in individuals suffering from other serious bacterial infections, including necrotizing fasciitis [42, 43]. Recently, elevated levels of endogenous melatonin were found to correlate with the ability to resist brucellosis infection [44]. As observed by Snider et al. [45] in COVID-19-infected patients, excessive blood levels of sPLA2-IIA (a toxic phospholipase; see below) due to a bacterial infection are consequential in causing lung surfactant changes culminating in acute lung injury [46].

As with both gram-negative and gram-positive bacterial infections inducing sepsis, likewise fungal infestations, which are highly detrimental to lung physiology and difficult to successfully treat, promote similar organ and systemic pathologies, including sepsis and all of its ramifications [47, 48]. As highlighted by Root-Bernstein [12], the worst-case scenario for developing serious COVID-19 disease may involve combined infection of the lungs with the SARS-CoV-2 virus and bacteria/fungi causing a synergistic action in stimulating an exaggerated cytokine storm and inducing acute respiratory distress syndrome (ARDS). Since melatonin is proposed to reduce the severity of viral, bacterial, and fungal infections, in the model proposed by Root-Bernstein [12], melatonin would seem to be a good treatment choice.

## Melatonin/HIF1α interactions

A hallmark of septicemia, regardless of whether it is caused by a bacterium, virus, or a fungus, is the conversion of the metabolic phenotype of activated immune cells from mitochondrial oxidative phosphorylation to cytosolic glycolysis, i.e., Warburg-type metabolism [49–52]. Major contributors to the switch from mitochondrial glucose oxidation to the upregulation of pyruvate metabolism in the cytosol often are accompanied by stimulation of hypoxia-inducible factor-1α (HIF-1α) and activation of NF-ҝB and other transcription factors which exaggerate inflammation [53–55]. In COVID-19-infected patients, macrophages convert from M2 anti-inflammatory to M1 pro-inflammatory cells which, along with other immune cells that exhibit a similar switch, contribute to the cytokine storm which leads to serious damage not only to the respiratory system but also in many other organs, thereby precipitating multiple organ failure [44, 56] (Fig. 1). Thus, melatonin may reduce the damage resulting from COVID-19-mediated septicemia by quelling HIF-1α, suppressing NF-ҝB, inhibiting the inflammasome and converting pro-inflammatory M1 macrophages to anti-inflammatory M2 macrophages while also reversing Warburg-type metabolism [57, 58] (Fig. 1). The negative role of inducible factor-1α (HIF-1α) activation has been mentioned as a contributing factor to serious SARS-CoV-2 infections which involve the upregulation of the cytokine storm and the associated multi-organ failure [59–62].

HIF1-α, also known as oxygen sensing transcription factor, is activated as a result of systemic hypoxia which occurs after the accumulation of edema and cellular debris in the respiratory alveoli which leads to poor O2/CO2 exchange. Increased mortality is observed in patients with elevated HIF-1α and an associated severe cytokine release [63]. Incremental changes in the level of HIF-1α also help to explain why diabetics are more prone to severe SARS-CoV-2 infections [64]; hyperglycemia leads to increased glycolysis in inflammatory monocytes and macrophages. This facilitates viral replication which contributes to elevated reactive oxygen species (ROS) generation which, in turn, stabilizes HIF1-α. This supports glycolysis and exaggerates the cytokine storm in these individuals.

Melatonin is a well-recognized suppressor of HIF-1α under a number of experimental conditions [65–67]. While the mechanism of that suppression is not specifically known [68], it may be the result of its direct inhibition of the cytosolic oxygen sensor or related to the potent antioxidant activity of the molecule which removes the agents that stabilize HIF-1α, i.e., ROS [69]. Thus, if the prediction of Codo et al. [64] is valid, which we feel it is, the inhibition of HIF-1α would contribute to a reduction in lung damage and COVID-19 severity, then the use of melatonin as a treatment of SARS-CoV-2-infected patients would be further justified [70–72].

## Melatonin/PLA2 interactions

The recent discovery of a correlation between circulating secreted phospholipase-A2 (Group IIA) (sPLA2-IIA) and the severity of COVID-19 disease [45] prompted the consideration of another potential mechanism by which melatonin may inhibit this viral infection. What Snider et al. [45] reported is that sPLA2-IIA levels and blood urea nitrogen (BUN) concentrations, indicative of renal damage, were strongly associated with the intensity of the infection and mortality of the infected patients. As a result of these correlations, the authors used the PLA-BUN index as a means to evaluate the likelihood of severe COVID-19 infections. Moreover, they proposed that suppression of sPLA2-IIA levels could be an important target to prevent or attenuate multiple organ failure, disseminated intravascular coagulation, and death of SARS-CoV-2-infected subjects.

Activated sPLA2-IIA is an inflammatory agent that is particularly destructive to cell biomembranes due to its ability to hydrolyzed fatty acids [73–75]. The membrane damage leads to the release of arachidonic acids and lysophospholipids; the arachidonic acids are then metabolized by cyclooxygenase to thromboxanes, prostacyclins, prostaglandins, etc. These eicosanoids promote inflammation and oxidative stress in multiple organs [76–78], which significantly contributes to vital organ dysfunctions (Fig. 1). The cell membranes that are damaged under systemic hyperinflammatory conditions include those of the mitochondria with energy failure being commonplace in these diseased situations [79]. By inhibiting cyclooxygenase, melatonin attenuates the hyperinflammatory response that accompanies a SARS-CoV-2 infection [80]. Hyperinflammation is often also accompanied by metabolic reprogramming, i.e., conversion of the inflamed cells to a usually pathological Warburg-type metabolism [81], a process that is reversed by melatonin [82, 83].

In addition to being elevated in individuals infected with SARS-CoV-2, high sPLA2-IIA levels have been measured in patients suffering from bacterial sepsis, a major killer of humans throughout the world, as well as from cardiac and hemorrhage shock [84]. The toxic actions of snake venoms also involve, along with an elevation of metalloprotease activities, increases in PLA2 [85]. Symptoms of snake venom toxicity include out-of-control inflammation and coagulopathy along with other complications and metabolic reprogramming of macrophages to the M1 hyperinflammatory phenotype [86]. In this context, the ability of melatonin to mitigate the toxicity of snake venoms has been documented [87, 88]. Although neither of these studies specifically examined the actions of melatonin on PLA2 activity, they report that it did counteract the extensive oxidative damage and inflammation in animals that were injected with the venom of the Egyptian cobra (*Naja haje*) or that of the saw scaled viper (*Echis carinatus*). The other enzymes known to be upregulated as a result of a venomous bite are metalloproteinases which in other pathological situations are also inhibited by melatonin [89]. Additionally, melatonin counteracts the effects of the nematocyst toxin of the purple-striped jelly fish (*Pelagia noctiluca*) [90]. The composition of this toxin is not well characterized but includes neuropeptides, prostanoids, membrane pore forming toxins, etc.

The commonality among the symptoms of these different conditions as they relate to sPLA2-IIA may explain some of the beneficial actions of melatonin as observed in a variety of disease situations, especially when intensive inflammation, excessive oxidative stress, mitochondrial dysfunction, metabolic reprogramming, and so on are involved. These signs are all common in individuals infected with SARS-CoV-2, where melatonin is protective. Melatonin has an unusually diverse skill set for suppressing diseases that are associated with these changes. Melatonin is a confirmed potent anti-inflammatory agent and antioxidant in many experimental models and clinical situations [32, 91–94]. It has been widely suggested for use as an antidote to COVID-19 infections, a recommendation supported by studies in both animal and in human studies [95, 96]. Clinically, melatonin treatment reduces the severity of SARS-CoV-2 infections in terms of lowering the seriousness of symptoms, decreasing the need for hospitalization (which simultaneously helps control health care exhaustion), reducing the duration of hospital stay when this is necessary, eliminating the need for mechanical intubation, and lessening the death rate [4, 11]. Even prior to the identification of the current COVID-19 pandemic, melatonin was shown effective in reducing oxidative damage and lowering the inflammatory burden in other viral diseases [97–101], including those caused by other coronaviruses [102], and it has been classified as a potential pan-antiviral agent, although it is not viricidal.

## Effective dose, timing of administration, limitations, and future studies

Applications have been made for 10 clinical trials; most of these are on-going and are summarized in tabular form in the comprehensive report of Ramos et al. [16]. In these trials, total melatonin doses ranging between 2 mg daily and 500 mg daily are proposed for use; these are either given orally once per day or equally divided in multiple doses over a 24-h period. One study is designed to use intravenously infused melatonin. The adult patients selected for treatment range from newly diagnosed to critically ill subjects in intensive care units; the primary outcomes to be assessed vary widely among the trials. None of these studies have proposed the use of children, possibly because SARS-CoV-2 infections are less common in young individuals. As in adults, melatonin use and safety in children has been tested where high doses of melatonin have been proven safe [41].

The wide variety of melatonin doses proposed in these trials is much like the already-published reports on the use of melatonin to treat COVID-infected adults (Table 2). In these reports the amounts of melatonin given fall between 3 and 600 mg daily. Whereas all the studies reported positive outcomes when melatonin was used, none observed any toxicity of melatonin, including at the highest doses employed. In the report of a network analysis of drugs potentially useful in treating COVID, melatonin was suggested as the one to have the greatest likelihood of controlling SARS-CoV-2 infections [11]. The dose of melatonin that may be effective would possibly vary according to the age of the infected patient, since in aged individuals melatonin levels are often greatly diminished [106, 107]. Indeed, the greater susceptibility of the elderly to a SARS-CoV-2 has been speculated to be a result of the reduction in endogenous melatonin production [108]. Likewise, body size may be a consideration regarding the amount of melatonin administered. As a general rule, perhaps 1 mg per kg body weight may be a starting point. Due to the high safety profile of melatonin, subsequent trials should also include individuals over a wide age range, including children.

**Table 2:** Clinical studies/trials in which melatonin was tested as a treatment for SARS-CoV-2 infection

Reference	Study type	Number of patients	Total dose of melatonin	Outcomes
Castillo et al [95]	Retrospective case	20	Oral, 36–72 mg (4 doses)	↓Need for mechanical ventilation ↓Duration of hospitalization ↑Survival
Farnoosh et al. [96]	Randomized double-blind	44	9 mg (3 doses)	↓Pulmonary symptoms ↓CRP* ↓Duration of hospitalization
Hassan et al. [103]	Randomized prospective	158	10 mg	↓Sepsis ↓Microvascular coagulation ↓Mortality
Mousavi et al. [104]	Randomized prospective	96	3 mg	↑Blood oxygen ↑Sleep time
Alizadeh et al. [105]	Randomized prospective	31	6 mg	↓COVID symptoms ↓CRP
Ramlall et al. [4]	Retrospective	13, 394	Different doses	↓Need for intubation ↑Outcome for those intubated

The studies varied widely in terms of melatonin dosage and endpoints measured. Each of the reports suggest melatonin has efficacy in improving the outcome of the infected patients. The readers are urged to consult the original publications for further details

^*^CRP C-reactive protein

When melatonin is used as a sleep aid or for some other uses, it is typically taken just before bedtime. This also is consistent with the rise in the nighttime endogenous melatonin released from the pineal gland [109]. In extreme situations, such as serious illness as during a COVID-19 infection (Table 2), melatonin was sometimes given in divided doses throughout each 24-h period. The rationale for this is that the elevated free radical generation as well as the inflammatory response associated with this disease persists during both the light and dark period. To aid in the inhibition of these damaging responses, it may improve the clinical outcome if melatonin is used as a treatment of infected individuals during the day, when circulating melatonin levels are at their nadir [109, 110].

There are some limitations to the already-published studies. While the endpoints were generally objective in terms of their measurement, usually the studies were not blinded. Co-morbidities among the treated patients varied as did the other drugs that were used concurrently. Some of the studies included a small number of patients. At this point, there is no standardization of the optimal route of administration or of the time of day that would yield the best results. The most effective or necessary dose is also not established. On the positive side, melatonin has essentially no toxicity and, as a result, no LD50 has been established despite attempts to do so. As much as 1000 mg has been given to healthy humans every day for a month without any substantial negative effects [111]. Also, melatonin is safe when administered via multiple routes. A key observation in a couple of the clinical reports was the reduced mortality of the melatonin-treated subjects (Table 2). Considering the preliminary findings in humans and the very large experimental base of information that strongly supports its efficacy as a treatment of COVID-19, well-designed, placebo-controlled, double-blinded studies are needed. Hopefully, the on-going approved clinical trials will resolve essential treatment issues. Meanwhile, steps to identify in greater detail the mechanisms by which melatonin resists this viral and other viral infections should continue.

## Concluding remarks

Beyond melatonin’s well-known antioxidant and anti-inflammatory actions which have proven the efficacy of this molecule in the treatment of diseases/ conditions where excessive free radical-mediated oxidative damage and hyperinflammation are causative factors [112–116], the studies summarized herein also support its use as a possible treatment for COVID-19 disease. Melatonin has been proposed as a potential effective inhibitor of the destructive inflammatory consequences of a SARS-CoV-2 infection [2, 4, 7, 11], an idea supported by observed and predicted improvements in the outcome of patients with this disease [95, 96] (Table 2). Numerous inter-related factors conspire to enhance the cytokine storm and multiple organ failure associated with COVID-19 disease severity and mortality, including elevated sPLA2-IIA, development of pro-inflammatory M1 macrophages, activation of HIF-1α, conversion to Warburg-type metabolism of immune cells, damage to mitochondria, massive release of cytokines, oxidative stress, etc. [117–120] (Fig. 1); each of these actions have been shown to be counteracted by melatonin. A center piece of this series of processes may be the alterations in mitochondrial physiology and the shift of glucose oxidation to the cytosol. This change in glucose handling markedly alters the metabolism of the mitochondria, which is critical to limiting cellular dysfunction, resisting disease, and preventing organismal death. Indeed, there are numerous maladies that are specifically classified as mitochondria-related diseases [121–125] with this category, including viral infections, such as SARS-CoV-2 [126–129].

If melatonin is in fact a significant antidote to SARS-CoV-2 infection, the development of a Warburg-type metabolism by hyperactive immune cells and other diseased cells [130], may be indirectly a major contributor to COVID-19 disease. This is because when intracellular glucose metabolism is reprogrammed from the mitochondria into the cytosol, the mitochondria can no longer synthesize acetyl coenzyme A (acetyl-CoA). This has high importance, since acetyl-CoA is a required co-substrate for intramitochondrial melatonin production [131], which normally occurs in these organelles of healthy cells but likely not in the mitochondria of highly inflamed cells [129]. Thus, in the absence of local melatonin synthesis in infected cells, the loss of this locally produced potent endogenously generated anti-inflammatory and antioxidant agent, the mitochondria lose a major portion of their protection against reactive oxygen species, inflammatory cytokines, etc., leading to their dysfunction; this contributes to a weakening of the cells with an increased susceptibility to cellular destruction by SARS-CoV-2. This would help explain the published data documenting the ability of melatonin to resist virus-related diseases, including that related to several different coronaviruses [102]. The ability of melatonin to reverse the Warburg effect in pathological cells in humans was recently documented, presumably allowing the mitochondria also to synthesize melatonin [129, 130].

The failure of melatonin to attract attention as a potential treatment for COVID-19 is somewhat disappointing considering a number of scientific/medical papers that have recommended its use. This may relate to a number of factors, including the lack of promotion of its therapeutic use for this disease by any influential group. Numerous already-available pharmaceutical drugs have been repurposed for the potential treatment of COVID-19. Yet, no organization/agency has proposed the use of melatonin even though it is much less expensive (sometimes a 100-fold less costly than the proposed prescription medications), and based on the outcomes of recent published trials [95, 96], it has efficacy in treating this condition. After an analysis of 27 publications related to the ability of drugs to successfully treat COVID-19, the authors concluded that melatonin is at least twice as effective as remdesivir or tocilizumab in reducing the inflammatory markers of a coronavirus 2019 infection [132]. Both remdesivir (Veklury) and tocilizumab (Actemra) are FDA authorized for use to treat select COVID patients suffering with a severe infection; both drugs have notable side effects and are given intravenously [133, 134]. In contrast, melatonin has a high safety profile and can be taken orally or administered by any other route [16]. Since melatonin is non-patentable and is inexpensive, the incentive of the pharmaceutical industry to support its use is lost. Finally, pharmaceutical drugs are sometimes enthusiastically advanced by individuals who stand to gain financially [135].

Better means of treatment for COVID-19 and other diseases, especially when a medication is less expensive and the toxicity of the suggested drug is minimal. All reasonable treatment options should equally be considered, not only those that have the backing of the most influential medical/pharmaceutical personnel [136]. Some in the profession have considered the COVID-19 pandemic an opportunity that should be exploited for personal gain. This is not permissible in medicine. There are examples of bias and/or conflicts of interest when treatment options for COVID-19 are considered [137].

A major purpose of the current report is to urge “leveling of the playing field” such that all potential reasonable options be considered to fight any serious, rapidly spreading disease, not only the COVID-19 pandemic [138]. The World Health Organization made a similar claim in 2014 during the Ebola outbreak in western Africa. In grave crises, such as during an Ebola epidemic or the COVID-19 pandemic, it is ethical to take advantage of all possible available and safe treatments even if their efficacy may not be definitively established especially when the drug has no serious side effects. Indeed, considering a large number of deaths that continue to occur worldwide due to SARS-CoV-2 infections, it may be unethical not to take advantage of any potentially safe treatments, especially if the vaccines become less effective due to continued mutations of the virus. People who are vulnerable and may be infected with such diseases should not have to wait for the development of a new vaccine which often requires months to years, an interval during which death of many patients may be the outcome. Additionally, the currently available mRNA COVID-19 vaccines are not safe for everyone, in particular for those who may have multiple allergies, and many individuals refuse to be vaccinated [139–141]. Moreover, the vaccines are not universally protective since vaccinated individuals still die of SARS-CoV-2 infections. The use of melatonin would be especially advantageous because it can be orally self-administered, it is low in cost, and it lacks significant toxicity. This applies especially to impoverished regions of the world where the populace has fewer financial resources to devote to the treatment of this disease and where health care is not readily available. Additionally, although this paper considers melatonin as a sole treatment for SARS-CoV-2 infections, it has also been suggested as a co-treatment with vaccines to improve their efficacy [15, 142–144] and in combination with other drugs [132]; this latter suggestion would be especially applicable when the medications have different but complimentary mechanisms of action to those of melatonin. Finally, to avoid or to reduce the likelihood of pervasive viral infections (e.g., by the highly deadly zoonotic Nipah virus currently invading India or the Omicron variation of SARS-CoV-2)) in the future, the profession should be more proactive as opposed to being reactive.

## Data Availability

Not applicable.
